# Development of a Fully Automated Graf Standard Plane and Angle Evaluation Method for Infant Hip Ultrasound Scans

**DOI:** 10.3390/diagnostics12061423

**Published:** 2022-06-09

**Authors:** Tao Chen, Yuxiao Zhang, Bo Wang, Jian Wang, Ligang Cui, Jingnan He, Longfei Cong

**Affiliations:** 1 Department of Ultrasound, Beijing Jishuitan Hospital, The 4th Clinical College, Peking University, Beijing 100035, China; aneeea@163.com; 2 Shenzhen Mindray Bio-Medical Electronics Co., Ltd., Shenzhen 518057, China; zhangyuxiao@mindray.com (Y.Z.); wangbo@mindray.com (B.W.); wangjian_d@mindray.com (J.W.); 3 Department of Ultrasound, Peking University Third Hospital, Beijing 100191, China; cuijuegang@126.com

**Keywords:** developmental dysplasia of the hip (DDH), ultrasound image, standard plane detection, computer-aided diagnosis, deep learning

## Abstract

Background: Graf’s method is currently the most commonly used ultrasound-based technique for the diagnosis of developmental dysplasia of the hip (DDH). However, the efficiency and accuracy of diagnosis are highly affected by the sonographers’ qualification and the time and effort expended, which has a significant intra- and inter-observer variability. Methods: Aiming to minimize the manual intervention in the diagnosis process, we developed a deep learning-based computer-aided framework for the DDH diagnosis, which can perform fully automated standard plane detection and angle measurement for Graf type I and type II hips. The proposed framework is composed of three modules: an anatomical structure detection module, a standard plane scoring module, and an angle measurement module. This framework can be applied to two common clinical scenarios. The first is the static mode, measurement and classification are performed directly based on the given standard plane. The second is the dynamic mode, where a standard plane from ultrasound video is first determined, and measurement and classification are then completed. To the best of our knowledge, our proposed framework is the first CAD method that can automatically perform the entire measurement process of Graf’s method. Results: In our experiments, 1051 US images and 289 US videos of Graf type I and type II hips were used to evaluate the performance of the proposed framework. In static mode, the mean absolute error of α, β angles are 1.71° and 2.40°, and the classification accuracy is 94.71%. In dynamic mode, the mean absolute error of α, β angles are 1.97° and 2.53°, the classification accuracy is 89.51%, and the running speed is 31 fps. Conclusions: Experimental results demonstrate that our fully automated framework can accurately perform standard plane detection and angle measurement of an infant’s hip at a fast speed, showing great potential for clinical application.

## 1. Introduction

Developmental dysplasia of the hip (DDH) is one of the most common disorders seen in infants, and its incidence ranges from about 0.1% to 3% depending on the population [1,2,3]. This condition represents a spectrum of mild dysplasia, to subluxation, to dislocation of the hip joint. The pathogenesis of DDH is multifactorial, involving both genetic and intrauterine factors [4]. The importance of identifying and treating DDH in the early stage is globally accepted, and research found that the incidence of late-diagnosed hip dislocation decreased from 0.9 to 0.12 per 1000 live births following an introduction of a screening program [5]. The treatment of DDH includes dynamic splinting and static splinting [6], and the Pavlik harness is the most popular one in early treatment, with the success rate ranging from 50% to 96% [7,8]. Left untreated, DDH may lead to serious consequences for patients, impacting their whole life. It is reported that DDH accounts for 10~15% of hip replacements in patients under 50 years of age [9]. Due to its ability to visualize cartilaginous hip structures and its advantages of providing real-time imaging and being non-ionizing radiation, non-invasiveness, and low cost, 2D ultrasound has become the gold standard technique for early diagnosis of DDH [10].

**Figure 1:**
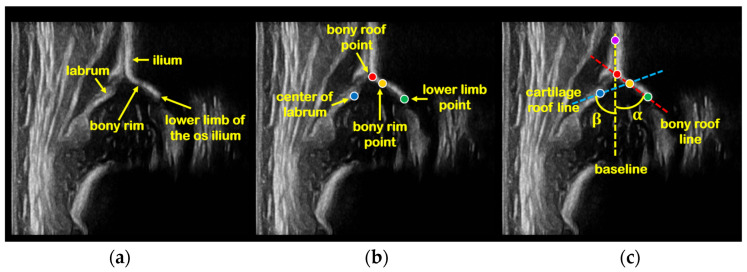
Example of a hip joint US image and Graf’s method. (**a**) The anatomical structures in standard plane. (**b**) Four landmarks determined based on the anatomical structures, bony roof point (red dot), bony rim point (yellow dot), lower limb point (green dot), and the center of labrum (blue dot). (**c**) Measurement lines and α, β angles. First, the uppermost point of the cartilaginous roof (purple dot) must be sought. From this pivot at the uppermost point of the cartilaginous roof, the baseline (yellow dash line) is a tangent placed cranial to caudal along the echo of the ilium. The lower limb of the os ilium (green dot) is the pivot point, and the bony roof line (red dash line) is a tangent placed laterally from the pivot point just touching the bony roof (red dot). The cartilage roof line (blue dash line) is drawn from the bony rim (yellow dot) through the center of the labrum (blue dot). α is the angle between bony roof line and baseline, and β is the angle between cartilage roof line and baseline.

Graf’s method [11,12] is the most common 2D US-based DDH analysis used in clinical practice, it can be summarized as two steps: (1) first, a standard plane from US video is determined according to the shape and positional relationship of the ilium, bony rim, lower limb of the os ilium, labrum, and other anatomical structures; (2) then, landmarks based on the anatomical structures are identified, and the α, β angles are measured and the hip joint is classified, as shown in Figure 1.

Although Graf’s method has been widely adopted clinically, there are still some problems regarding its application and promotion: (1) the identification of tiny structures, such as the bony rim point, is required in the process of standard plane selection, which is laborious and time-consuming, and the results are also inaccurate and subjective [13]. (2) There is high intra- and inter-observer variability of angle measurement [14], where the standard deviation may reach 3° for α and 6° for β, even when measured only by experienced sonographers [15]. (3) Measurement accuracy is highly correlated with the sonographers’ qualifications [16]. To deal with these problems, a large number of computer-aided diagnostic (CAD) methods have been proposed to reduce manual intervention and improve the quality and efficiency of the DDH diagnostic process. As shown in Table 1, most of the CAD methods focused on one of the following steps in the Graf diagnosis process, such as the evaluation of the standard plane [17,18,19,20,21,22,23], the measurement of α and β [15,24,25,26,27,28,29], or the classification of hip dysplasia severity [30,31]. Due to their powerful ability to hierarchically mine and utilize multi-level features, deep learning-based methods can generally bring more accurate and robust diagnostic results than conventional traditional image processing-based methods. However, for all of the methods shown in Table 1, computer assistance is provided for only part of the process, so they are semi-automated diagnostics solutions in essence. 

In this study, aiming to minimize the manual intervention in the diagnosis process, we developed a deep learning-based computer-aided framework for the DDH diagnosis, and it can perform fully automated standard plane detection and angle measurement for Graf type I and type II hips. The framework is composed of three novel modules: an anatomical structure detection module, a standard plane scoring module, and an angle measurement module. These modules utilize the deep object detection algorithm and traditional image processing technology. The proposed framework can run in two modes. In static measurement mode, our framework directly performs accurate angle measurement based on the standard planes given by sonographers, which is the most common paradigm of the current CAD methods. Furthermore, we innovatively support the dynamic measurement mode for the first time, and in this mode, our framework can automatically perform the two steps of Graf’s method without any manual intervention. First, each frame of the video is quantitatively evaluated according to the visibility, shape, and relative position of anatomical structures, and the frame with the highest score is then selected as the standard plane. Second, the α, β angles are automatically measured based on the selected plane. The framework can also report the Graf type (type I or type II) of the hip according to the α angle in both measurement modes. To the best of our knowledge, our proposed framework is the first CAD method that can automatically perform the entire measurement process of Graf’s method.

## 2. Materials and Methods

### 2.1. Data Acquisition 

This study was approved by the ethics committee of Beijing Jishuitan Hospital (protocol code 201805-09). To ensure the quality of data, the sonograms were obtained by two qualified and certified sonographers with 7–10 years of experience in infant hip screening. Both of them had attended a hip ultrasonography course given by Professor Graf. The exclusion criteria were as follows: (1) the infant twisted or did not cooperate; (2) US data with infant hip dysplasia caused by purulent hip arthritis, joint contractures, cerebral palsy, and other diseases were excluded; (3) US data of patients with other hip joint diseases and limb deformities were excluded; (4) US data with infant hips of Graf type D, type III, and type IV were excluded. From June 2019 to May 2020, they collected 2223 US images of Graf’s standard plane (1729 Graf type I and 494 Graf type II) and 289 US videos (238 Graf type I and 51 Graf type II) from 1341 infants. US images were collected from each infant, and US videos were collected from 173 infants. The life ages of the infants ranged from 0 to 6 months when they had the DDH ultrasound examination. The minimum age of the infants was 3 days, the maximum age was 168 days, and the median age was 87 days. A total of 510 infants (38.03%) are male, and the rest 831 infants (61.97%) are female. All US images or videos were captured by the Mindray Resona 7 ultrasound system with an L9-3U 3~9 MHz linear array transducer. For each US video, one sonographer first selected and saved a US image of Graf’s standard plane, and the image was then reviewed by the other sonographer. For each US image, one sonographer first annotated the bounding boxes of ilium, labrum, bony rim, and lower limb of the os ilium, then measured α, β angle with integer values according to Graf’s method, and finally confirmed the Graf type of hip joint. The results were also reviewed by the other sonographer. Both sonographers agreed with the final results in all cases.

### 2.2. The Fully Automated DDH Diagnosis Framework 

**Figure 2:**
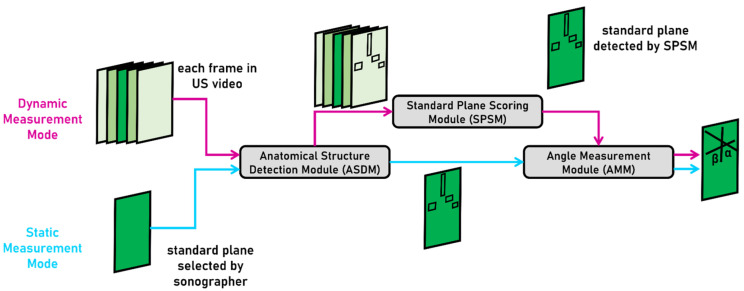
Overview of the proposed framework architecture. The upper route with purple marks is the workflow of DM mode and the lower route with blue marks is the workflow of SM mode.

We developed a deep learning-based computer-aided framework for the DDH diagnosis, and it can perform fully automated standard plane detection and angle measurement for Graf type I and type II hips. This framework utilizes deep object detection algorithm and traditional image processing technology and is composed of three modules: an anatomical structure detection module (ASDM), a standard plane scoring module (SPSM), and an angle measurement module (AMM). Figure 2 shows the framework architecture. The proposed framework can work in two modes, static measurement mode, and dynamic measurement mode, and the detailed procedures of each mode are described below:

- Static measurement mode (SM mode): Based on the standard plane US image selected by sonographer, ASDM is first used to detect four key anatomical structures in this image: ilium, labrum, bony rim, and lower limb of the os ilium. AMM is then used to generate landmarks followed by measurement of α, β angles.
- Dynamic measurement mode (DM mode): ASDM is first used to detect the four key anatomical structures in each frame of a hip US video, and SPSM is then used to quantify the quality of each frame according to the scoring formula we designed, and finally, AMM is used to measure α, β angles of the highest-scoring frame.

#### 2.2.1. Anatomical Structure Detection Module

**Figure 3:**
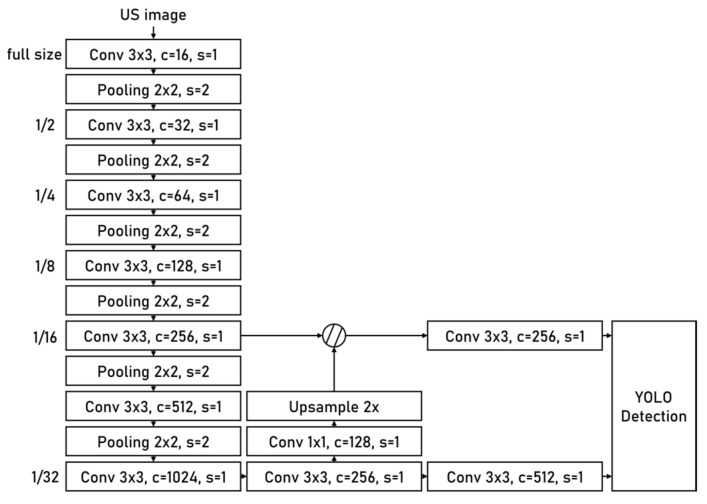
Overview of the YOLOv3-tiny network.

YOLOv3-tiny [32] is the main part of the anatomical structure detection module (ASDM). It is a lightweight one-stage object detection network exhibiting an excellent balance between inference speed and accuracy. Figure 3 shows the architecture of YOLOv3-tiny, which is composed of one backbone network and two branch networks. The backbone is built with 7 convolutional layers and 6 pooling layers and is used to extract features from the input image. Branch1 and branch2 are connected to conv5 and conv7, respectively, they implement object detection at 1/16 and 1/32 spatial scales. The output of the entire network is the summary of branch1 and branch2 results. There are two advantages of the dual-branch and multi-scale network design. First, it enhances the ability of the network to detect objects of different scales in the same image. Second, it increases the robustness of network when dealing with US images of different magnification factors. 

**Figure 4:**
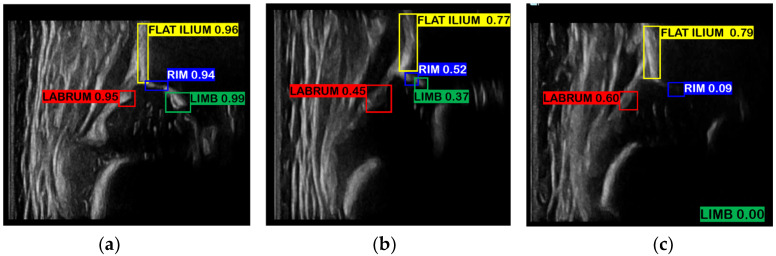
The network detection results. (**a**) Graf’s standard planes, where the anatomical structures are clear and visible for recognition, and can be accurately localized by the network, so they have high confidence scores. (**b**,**c**) Non-standard planes with unclear structures and low confidence scores. The lower limb of the os ilium cannot be recognized in (**c**), and thus the confidence score is 0.

In this study, the input of network is hip US images of size 416 × 416, and the output is the coordinate information and confidence score of four bounding boxes. The four bounding boxes each cover ilium, labrum, bony rim, and lower limb of the os ilium, which are the essential anatomical structures for standard plane detection and angle measurement. A large number of standard plane images of hips, which were annotated and reviewed by experienced sonographers, were used to train the network. Thus, for the clearer anatomical structure, the network will output a higher confidence score, and for the unclear or invisible anatomical structure in the non-standard plane, the network will output a lower confidence score, as shown in Figure 4. The confidence scores can represent the standard level of a plane to a certain extent. From the raw network output, the ASDM will filter out the bounding boxes with the highest score in each category as the final result. The relative position between these bounding boxes will also be checked by ASDM to ensure it is consistent with the clinical practice such that the result can be used in the subsequent scoring and measurement process.

#### 2.2.2. Standard Plane Scoring Module

Standard planes which meet Graf’s criteria are the prerequisite for subsequent accurate measurement and diagnosis. For this purpose, we proposed the standard plane scoring module (SPSM). SPSM can quantitatively evaluate the standard level of each frame in US video using the scoring formula in DM mode and can select the highest-scoring frame of this video as the standard plane for subsequent tasks. Based on Graf’s method and other clinical prior knowledge, the scoring formula is defined as:

(1) $$S=\lambda_{1}\cdot S_{lower\;limb}+\lambda_{2}\cdot S_{labrum}+\lambda_{3}\cdot S_{bony\;rim}+\lambda_{4}\cdot S_{ilium},$$

where S is the total score of a frame. $S_{lower\;limb}$, $S_{labrum}$, $S_{bony\;rim}$, and $S_{ilium}$ are scores for the lower limb of the os ilium, labrum, bony rim, and ilium, respectively, and $\lambda_{1}$ to $\lambda_{4}$ are the corresponding score weights. Graf’s method requires that the lower limb of os the ilium, labrum, and ilium must be shown clearly in the standard plane. Additionally, we add the evaluation of the bony rim because the bony rim point is one of the landmarks used to determine the cartilage roof line, which means that the measurement of β angle is directly affected by the quality of the bony rim.

The confidence score for each structure has a high reference value for standard plane scoring task due to the high quality of training data and annotations. Thus, $S_{lower\;limb}$, $S_{labrum}$, and $S_{bony\;rim}$ are assigned with the corresponding confidence scores, and we set $\lambda_{1}=3$, $\lambda_{2}=1$, and $\lambda_{3}=1$. There are three reasons that we make SPSM focus more on lower limb of the os ilium than the other two anatomical structures. (1) In Graf’s method, the lower limb of the os ilium must always be seen except in markedly decentered hip joints, and no diagnosis should be made if it is not seen. (2) Due to its deepest location, the visibility of lower limb of the os ilium is the most vulnerable to the depth of penetration of the ultrasound probe. (3) The lower limb of the os ilium is associated with the measurement of α angle, which is crucial to the classification of Graf’s types.

**Figure 5:**
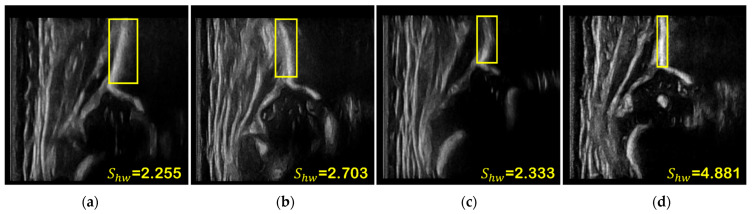
The effect of different ilium shapes on the height–width ratio of the bounding box. The ilium in (**a**,**b**) are inclined leftward and rightward, respectively. The ilium is curved in (**c**). The ilium is straight and vertical in (**d**), with the highest value for $S_{hw}$.

In the definition of Graf’s method, the straightness and tilt degree of ilium is critical for determining the standard level of a plane, but it is difficult to obtain this information by the confidence score only. Because the confidence score generally reflects more on the visibility of the ilium in a plane. For this reason, we established a scoring formula specifically for ilium:

(2) $$S_{ilium}=\lambda_{5}\cdot S_{conf}+\lambda_{6}\cdot S_{hw}+\lambda_{7}\cdot S_{angle},$$

where $S_{conf}$ is the confidence score of ilium, $S_{hw}$ is the height–width ratio of the bounding box of ilium, and $S_{angle}$ is the angle score of ilium. $\lambda_{5}$ to $\lambda_{7}$ are the corresponding weights of the above scores. As shown in Figure 5, $S_{hw}$ of a straight and vertical ilium is usually larger because it occupies less space in the width dimension, while $S_{hw}$ of a non-vertical or curved ilium is usually smaller. Therefore, it can be considered that, for different frames in one video, frames where ilium has a larger height–width ratio may be closer to the standard plane, and $S_{hw}$ is an indirect measure of the tilt degree of ilium by evaluating the shape of the bounding box. 

In addition, the angle θ between ilium and the vertical direction is measured according to image moment, which is a gray-scale distribution-based method [33]. The original box is first expanded to ensure that the bounding box contains the entire ilium as much as possible. We denote that the expanded box V is a rectangle region with I columns and J rows, and the p+q order image moments $M_{pq}$ can be expressed as follows:

(3) $$M_{pq}=\sum_{I}\sum_{J}i^{p}\cdot j^{q}\cdot V\left(i,j\right),$$

where p+q∈N. The coordinate of centroid is:

(4) $$\left\{x_{c},y_{c}\right\}=\left\{\frac{M_{10}}{M_{00}},\frac{M_{01}}{M_{00}}\right\},$$

and the θ can be calculated from the centroid, the zero-order moment, and the second-order moments:

(5) $$a=\frac{M_{20}}{M_{00}}-x_{c}^{2},b=\frac{M_{11}}{M_{00}}-x_{c}\cdot y_{c},c=\frac{M_{02}}{M_{00}}-y_{c}^{2},$$

(6) $$\theta =\frac{1}{2}\arctan\left(\frac{2b}{a-c}\right).$$

The angle score is designed based on the angle, which is defined as follows:

(7) $$S_{angle}=1-\left|\theta \right|/10.$$

The angle score directly and quantitively models the tilt degree of ilium. According to the definition, the more vertical the ilium is, the higher the angle score. $S_{angle}$ reaches the maximum value of 1 when θ is 0, and $S_{angle}$ decreases accordingly to penalize $S_{ilium\;}$ as θ increases. $S_{angle}$ will be negative if θ>10°, and this value of 10 can effectively deal with the measurement error of θ caused by random noise in the ROI, while ensuring the quality of the standard plane detection. The design of angle score can void the scoring system resulting in complete reliance on the deep learning network. We set $\lambda_{5}=0.2$, $\lambda_{6}=0.3$, and $\lambda_{7}=0.5$ according to their different importance in the process of ilium quality evaluation.

#### 2.2.3. Angle Measurement Module

We use maximum entropy threshold segmentation [34] to acquire the anatomical structures from the four bounding boxes’ output by ASDM. Then, gradient information and clinical prior knowledge are utilized to identify landmarks in the structures. The four landmarks are bony roof point, center of labrum, bony rim point, and lower limb point. The baseline is the tangent of uppermost ilium. The bony roof line is formed by bony roof point and lower limb point, and α is the angle between the bony roof line and baseline. The cartilage roof line is formed by bony rim point and the center of labrum, and β is the angle between the cartilage roof line and baseline.

### 2.3. Experiment Design 

The training process of the YOLOv3-tiny requires only static US images, in order to fully validate the performance of the proposed framework in DM mode, cases without US videos were randomly selected to construct training (565 cases, 938 US images) and validation (142 cases, 234 US images) sets. The remaining cases (634 cases, 1051 US images, and all 289 US videos) were used as the test set. Data augmentation was performed to expand the size of training set and improve the diversity of training data. The data augmentation operations include random scaling, random rotation, and Gaussian noise. There is a total of 19,698 images in the final training set.

The network was initialized using ImageNet [35] pre-trained weights, and the training process takes 30 epochs in total. The ADAM optimizer [36] was used with a learning rate of 1 × 10^−4^ and weight decay of 5 × 10^−4^. We saved the weights and validated the detection performance of the network at the end of each epoch. We used an early stopping strategy to determine which was the best performance model for subsequent tasks. 

We statistically analyzed the experimental results of the proposed framework in SM mode and DM mode. The software used was SPSS Statistics version 26 (IBM, Armonk, NY, USA). To validate the angle measurement performance of the framework, we used the standard deviation and mean absolute error (MAE) of the angle differences between the framework and sonographers to quantify the interobserver differences, and the intraclass correlation coefficient (ICC) and Bland–Altman plot were used to evaluate their agreement. The classification agreement rate (accuracy) and Cohen’s kappa coefficient were used to validate the classification performance of the framework. 

## 3. Results

### 3.1. Statistical Results of Static Measurement Mode

**Figure 6:**
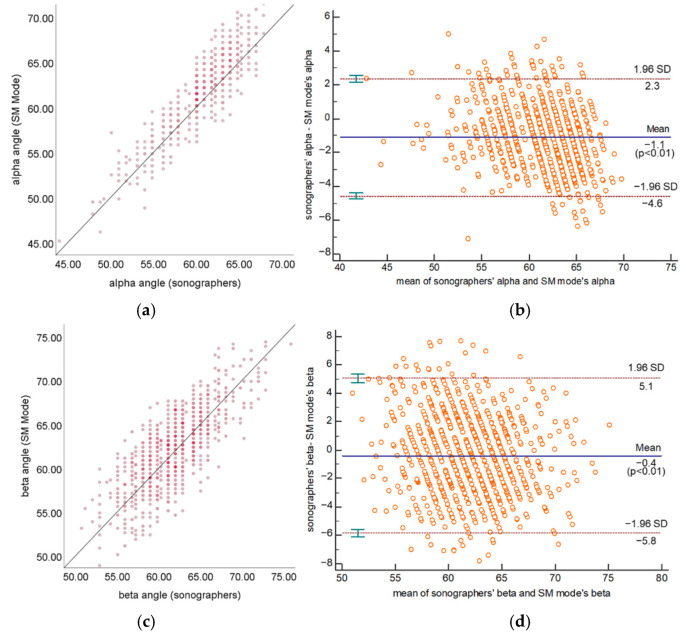
Scatter plots and Bland–Altman plots of α (**a**,**b**) and β (**c**,**d**) measured by SM mode and sonographers.

**Figure 7:**
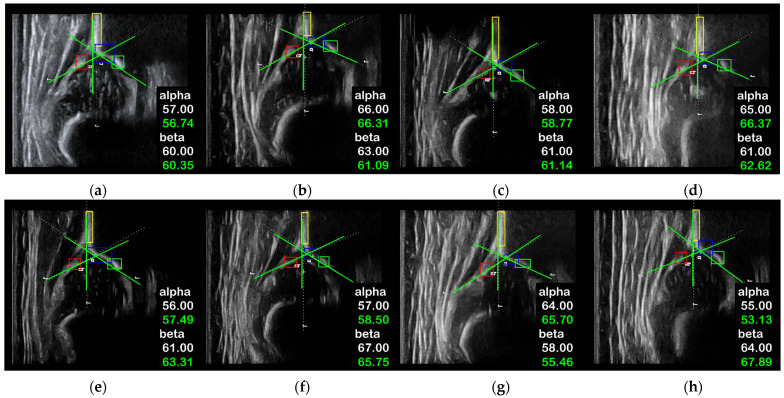
Qualitative comparisons for the angle measurement between the SM mode and the sonographers. The white dash lines are the manual plot in clinic. The green lines are formed by the SM mode based on the detected bounding boxes. The white values are given by sonographers, and the green values are given by the SM mode. The absolute differences between SM mode and sonographers are (**a**) Δα = 0.26°, Δβ = 0.35°; (**b**) Δα = 0.31°, Δβ = 1.91°; (**c**) Δα = 0.77°, Δβ = 0.14°; (**d**) Δα = 1.37°, Δβ = 1.62°; (**e**) Δα = 1.49°, Δβ = 3.31°; (**f**) Δα = 1.50°, Δβ = 1.25°; (**g**) Δα = 1.70°, Δβ = 2.54°; (**h**) Δα = 1.87°, Δβ = 3.89°.

A total of 1051 standard plane US images in the test set, including 798 type I hips and 253 type II hips, were used to test the measurement and classification performance of the proposed framework in SM mode. As summarized in Table 2, the standard deviation of the difference between SM mode and sonographers of α, β angles are 1.79° and 2.97°, respectively. The MAE of α, β angles are 1.71° and 2.40°. The ICCs of α, β angles are 0.85 and 0.73, respectively, both revealing good agreement. According to the Bland–Altman plots shown in Figure 6, the measurement results of α and β are symmetrically distributed around the reference line, and 95.46% and 96.29% of the measurement results of α and β, respectively, are within the limits of agreement. The classification accuracy of the SM Mode is 94.71%. Cohen’s kappa coefficient is 0.85, which shows good agreement. Figure 7 shows a comparison of some typical measurement results of the SM mode and the sonographers. 

### 3.2. Statistical Results of Dynamic Measurement Mode

**Figure 8:**
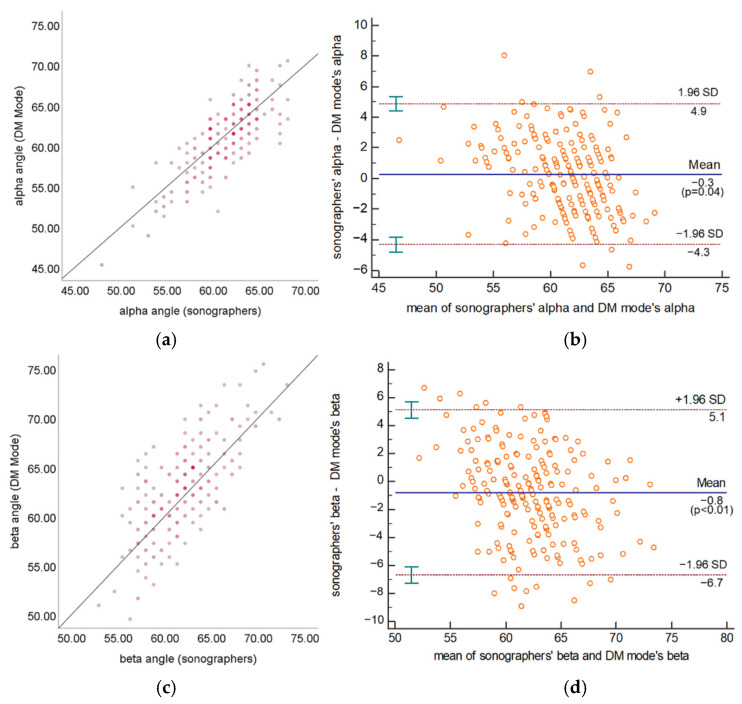
Scatter plots and Bland–Altman plots of α (**a**,**b**) and β (**c**,**d**) measured by DM w. SPSM and sonographers.

**Figure 9:**
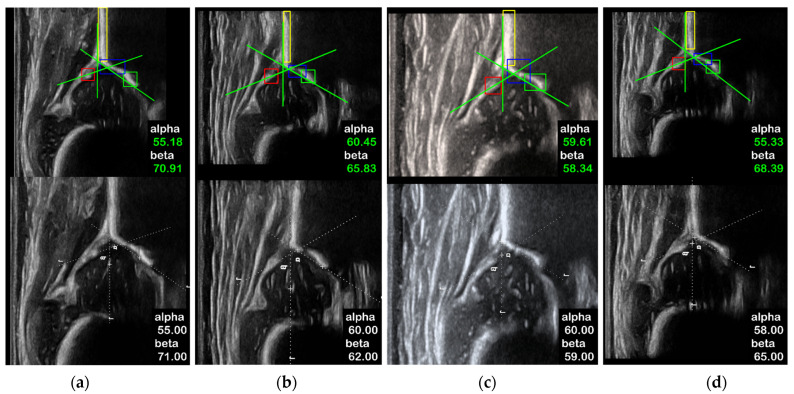
Qualitative comparisons on the angle measurement between the DM w. SPSM (**upper**) and sonographers (**lower**). The standard plane frames selected from the same US video are usually different, but the planes selected by DM w. SPSM and the angle measurement results are also reliable. The absolute differences between DM w. SPSM and sonographers are (**a**) Δα = 0.18°, Δβ = 0.19°; (**b**) Δα = 0.45°, Δβ = 3.83°; (**c**) Δα = 0.39°, Δβ = 0.64°; (**d**) Δα = 2.67°, Δβ = 3.39°.

A total of 289 US videos and their corresponding standard plane US images in the test set, including 238 type I hip joints and 51 type II hip joints, were used to test the measurement and classification performance of three modes: (1) the DM mode with SPSM (DM w. SPSM); (2) the DM mode without SPSM (DM w/o. SPSM), and using the sum of confidence scores as the scoring formula to evaluate planes; (3) the SM mode. We used these modes to validate the effectiveness of SPSM. As summarized in Table 3, the standard deviation of the difference between the DM w. SPSM and sonographers of α and β are 2.43° and 3.15°, respectively, and both are higher than the standard deviation measured by the SM mode (α: 1.66°, β: 2.91°), but much less than the DM w/o. SPSM (α: 3.30°, β: 4.29°). The MAE of α and β between the DM w. SPSM and sonographers are 1.97° and 2.53°, respectively, and both are higher than the values measured by SM mode (α: 1.59°, β: 2.28°), but much less than DM w/o. SPSM (α: 2.61°, β: 3.64°). For the DM w. SPSM, the ICCs of α and β are 0.80 and 0.68, respectively. They show less agreement compared with the values by SM mode (α: 0.85, β: 0.71), but show higher agreement compared with those by DM w/o. SPSM (α: 0.64, β: 0.44). According to the Bland–Altman plots shown in Figure 8, the measurement results of α and β are symmetrically distributed around the reference line, and 97.22% and 93.97% of the measurement results of α and β, respectively, are within the limits of agreement. Table 4 shows the classification performance of three modes, and the Cohen’s kappa coefficients of DM w/o. SPSM, DM w. SPSM, and SM mode are 0.39, 0.76, and 0.89, respectively. The classification agreement rates are 71.73%, 89.51%, and 95.80%. Figure 9 shows some typical measurement results of DM w. SPSM and sonographers. In summary, the angle measurement performance and classification performance of DM w. SPSM is between DM w/o. SPSM and SM mode, but much closer to SM mode.

### 3.3. Running Speed of Dynamic Measurement Mode

The running speed of the framework in dynamic measurement mode was tested using a hardware environment of an Intel Core i7-9700 CPU, 32 GB of RAM, and an NVIDIA GeForce 1660 GPU. The software on it is Windows 10 (Microsoft, Redmond, WA, USA), Python 3.6 (Python Software Foundation, Beaverton, OR, USA), and TensorFlow 1.8 (Google, Mountain View, CA, USA). The average speed of the plane scoring and angle measurement on 289 US videos is 31 fps. 

## 4. Discussion

In this study, we proposed a deep learning-based computer-aided framework for the DDH diagnosis of Graf type I and type II hips, which can not only perform the common static measurement mode like the current CAD methods but also innovatively realize the dynamic measurement mode for the first time. To the best of our knowledge, there is currently no CAD method that can automatically complete the entire measurement process of Graf’s method.

In the static measurement mode, the measurement and classification are directly performed based on the standard planes selected by a sonographer. On the test set, the standard deviation of the difference between SM mode and sonographers is lower than the standard deviation of the difference between human observers (α: 3.2°, β: 6.1°) reported by [37]. The ICCs of α and β in SM mode were higher than the ICCs between human observers (α: 0.72, β: 0.34) reported by [38]. The above results demonstrate that, in terms of measurement accuracy, the performance of SM mode is better than that of human observers. The MAEs of the α, β angles demonstrate that SM mode also performs better than other deep learning-based methods [28,29]. 

Since there are only Graf type I and type II hip joints in the dataset, we used 60° as the boundary and performed a binary classification according to α measured by the framework. It can be seen from Table 2 that the mean α value of the test set is 60.94°, which means that measurement error may lead to misclassification [28]. While in SM mode, both the classification agreement rate and Cohen’s kappa show high classification performance compared with that of experienced sonographers (classification agreement rate: 82%~91%, Cohen’s kappa: 0.60~0.86) reported by [38] and better than for other CAD methods [28,29,31]. Nevertheless, we still suggest sonographers pay more attention to the potential misclassification situation of hips with α around 60° (58°~62°).

**Figure 10:**
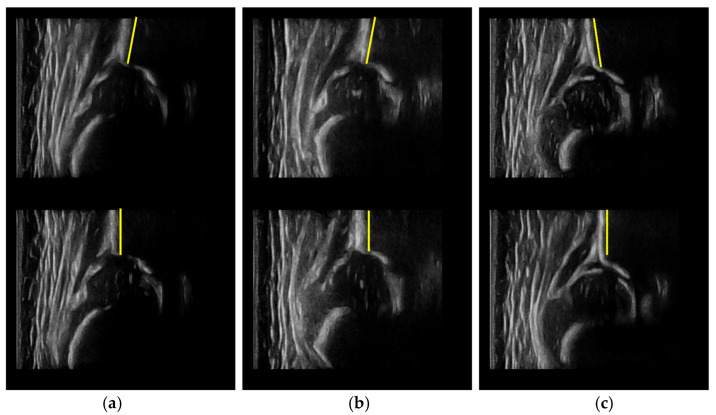
Comparisons of standard planes selected by DM w/o. SPSM (**upper**) and DM w. SPSM (**lower**). The ilia selected by DM w/o. SPSM are titled to the left (**a**,**b**), or the right (**c**), but the ilia selected by DM w. SPSM are all vertical and straight.

The dynamic measurement mode is an important innovation of this study. For the dynamic measurement mode, SPSM is the decisive factor for its measurement and classification performance, so we designed DM w. SPSM and DM w/o. SPSM to validate it. As shown in Table 3 and Table 4, compared with DM w/o. SPSM, DM w. SPSM can provide more accurate angle measurement results, the MAEs of α and β of DM w. SPSM are reduced by 0.64° and 1.11°, respectively, and the ICCs of α and β are much higher. Different angle error levels are reflected by the classification performance, where DM w. SPSM has much higher classification agreement rate and Cohen’s kappa. Although confidence scores from the network have a high reference value for the evaluation of the standard level of a certain plane, they are usually more related to the structure visibility and cannot properly indicate whether the shape of the anatomical structure meets the requirements. SPSM simultaneously takes confidence scores, the positional relationship between the anatomical structures, and the shape of the ilium into consideration, so the selected standard planes based on it are usually more reliable. The SPSM can identify the standard planes that have clear anatomical structures with correct positional relationships and straight and vertical ilium, as shown in Figure 10.

Although the SPSM can select standard planes with higher quality in DM mode, it is still difficult to guarantee that these are identical to planes selected by sonographers. This means that the measurement results of DM mode and sonographers exist inter-scan inter-observer discrepancies other than same scan inter-observer discrepancies of SM mode. In general, the former will lead to greater errors. As shown in Table 3 and Table 4, DM w. SPSM has a slightly greater standard deviation of differences and MAEs compared with SM mode and shows lower ICCs, though still higher compared to the ICCs between human observers [38]. The classification agreement rate and Cohen’s kappa of DM w. SPSM are similar to the classification agreement of human observers [37,38]. As shown in Figure 9, although the SPSM-selected plane is not identical to the sonographer-selected plane in the same video, it still meets Graf’s criteria and can produce reliable measurement results. In summary, the dynamic measurement mode of the proposed framework shows good measurement performance and classification performance, similar to experienced sonographers. In addition, compared with the methods based on two-stage instance segmentation networks [28,29], our YOLOv3-tiny-based method has more advantages in terms of the number of parameters, computational cost, and inference speed, which makes it possible to implement our framework in ultrasound devices. Considering that it is a fully automated diagnostic pipeline with high running speed and does not require any manual intervention, the application of the dynamic measurement mode may effectively improve the work quality and efficiency of sonographers in infant DDH screening.

Our study also has several limitations. First, we just performed a simple binary classification (Graf type I and type II) of the hips by comparing the α angle with 60° and did not utilize any age information to implement the differentiation of Graf subtypes. Second, the number of US videos used to test the DM mode is still small, so the generalization ability of DM mode may not have been sufficiently evaluated. In the future, we plan to take the week/month age information and β angle into account to implement the classification of Graf subtypes to better assist sonographers in the diagnosis of DDH. We will also collect more US videos and hip joint data with α angle under 50° (Graf type IIc) to further improve and evaluate the performance of the proposed framework.

## 5. Conclusions

In this study, we proposed a deep learning-based computer-aided framework for the DDH diagnosis of Graf type I and type II hips. It can not only run in the static mode like other CAD methods but also innovatively support the dynamic mode, which includes standard plane detection from ultrasound video and angle measurement. To the best of our knowledge, our proposed framework is the first CAD method that can automatically perform the entire measurement process of Graf’s method. The performance of both modes is comparable to that of human experts. The high running speed indicates that it can run in real-time as well. Therefore, our framework has great potential to assist sonographers in routine DDH screening.

## Figures and Tables

**Table 1:** Brief presentation of existing CAD methods for the diagnosis of DDH.

Aim	Type of CAD	Author	Year	Algorithm	Applicable Data
Standard Plane Evaluation	Conventional CAD	Quader et al. [17]	2021	random forest classifier	3D volume
		Hareendranathan et al. [18]	2021	manual method	2D standard plane
	Deep Learning-based CAD	Paserin et al. [19,20]	2017	CNN	3D volume
		Paserin et al. [21]	2018	LSTM	3D volume
		El-Hariri et al. [22]	2021	3D U-Net	3D volume
		Liu et al. [23]	2021	NHBS-Net	2D standard plane
Angle Measurement	Conventional CAD	Quader et al. [15]	2017	morphological and geometric features	2D standard plane
		Quader et al. [17]	2021		3D volume
		Sezer et al. [24]	2019	level set	2D standard plane
	Deep Learning-based CAD	Golan et al. [25]	2016	FCN	2D standard plane
		Hareendranathan et al. [26]	2016	CNN	2D standard plane
		El-Hariri et al. [27]	2019	2D U-Net	2D standard plane
		Hu et al. [28]	2021	multi-head Mask R-CNN	2D standard plane
		Lee et al. [29]	2021	Mask R-CNN + FCN	2D standard plane
Graf Type Classification	Deep Learning-based CAD	Sezer et al. [30]	2020	CNN	2D standard plane
		Gong et al. [31]	2021	DNN + random forest classifier	2D standard plane

**Table 2:** Comparison of angle measurement between SM mode and sonographers.

	Sonographers	Framework (SM Mode)	Framework-Sonographer	MAE	ICC (95% CI)	Agreement
α	60.94 ± 3.54	61.88 ± 4.08	1.09 ± 1.79	1.71	0.85 (0.84~0.87)	Good (>0.7)
β	61.57 ± 3.82	62.20 ± 4.26	0.44 ± 2.97	2.40	0.73 (0.70~0.76)	Good (>0.7)

**Table 3:** Comparison of angle measurement between DM w/o. SPSM, DM w. SPSM, SM mode, and sonographers.

		Angle Measurement	Difference	MAE	ICC (95% CI)	Agreement
sonographers	α	61.35 ± 3.26	–	–	–	–
	β	61.52 ± 3.51	–	–	–	–
DM w/o. SPSM	α	61.18 ± 3.41	–0.37 ± 3.30	2.61	0.64 (0.59~0.69)	Moderate (0.5~0.7)
	β	61.57 ± 3.70	1.55 ± 4.29	3.64	0.44 (0.38~0.51)	Poor (<0.5)
DM w. SPSM	α	61.04 ± 4.17	−0.31 ± 2.43	1.97	0.80 (0.75~0.84)	Good (>0.7)
	β	62.58 ± 4.35	0.80 ± 3.15	2.53	0.68 (0.61~0.74)	Moderate (0.5~0.7)
SM mode	α	62.31 ± 3.74	1.03 ± 1.66	1.59	0.85 (0.81~0.88)	Good (>0.7)
	β	61.95 ± 4.15	0.17 ± 2.91	2.28	0.71 (0.65~0.76)	Good (>0.7)

**Table 4:** Classification performance of DM w/o. SPSM, DM w. SPSM, and SM mode.

	Classification Agreement Rate (%)	Cohen’s Kappa	Agreement
DM w/o. SPSM	71.73	0.39	Poor
DM w. SPSM	89.51	0.76	Good
SM mode	95.80	0.89	Good

## Data Availability

The data are not publicly available due to the patient’s right to privacy.
